# Urbanization and health in China, thinking at the national, local and individual levels

**DOI:** 10.1186/s12940-016-0104-5

**Published:** 2016-03-08

**Authors:** Xinhu Li, Jinchao Song, Tao Lin, Jane Dixon, Guoqin Zhang, Hong Ye

**Affiliations:** Key Laboratory of Urban Environment & Health, Institute of Urban Environment, Chinese Academy of Sciences, Xiamen, 361021 China; National Centre for Epidemiology and Population Health, the Australian National University, Canberra, ACT 0200 Australia

## Abstract

**Background:**

China has the biggest population in the world, and has been experiencing the largest migration in history, and its rapid urbanization has profound and lasting impacts on local and national public health. Under these conditions, a systems understanding on the correlation among urbanization, environmental change and public health and to devise solutions at national, local and individual levels are in urgent need.

**Methods:**

In this paper, we provide a comprehensive review of recent studies which have examined the relationship between urbanization, urban environmental changes and human health in China. Based on the review, coupled with a systems understanding, we summarize the challenges and opportunities for promoting the health and wellbeing of the whole nation at national, local, and individual levels.

**Results:**

Urbanization and urban expansion result in urban environmental changes, as well as residents’ lifestyle change, which can lead independently and synergistically to human health problems. China has undergone an epidemiological transition, shifting from infectious to chronic diseases in a much shorter time frame than many other countries. Environmental risk factors, particularly air and water pollution, are a major contributing source of morbidity and mortality in China. Furthermore, aging population, food support system, and disparity of public service between the migrant worker and local residents are important contributions to China’s urban health.

**Conclusions:**

At the national level, the central government could improve current environmental policies, food safety laws, and make adjustments to the health care system and to demographic policy. At the local level, local government could incorporate healthy life considerations in urban planning procedures, make improvements to the local food supply, and enforce environmental monitoring and management. At the individual level, urban residents can be exposed to education regarding health behaviour choices while being encouraged to take responsibility for their health and to participate in environmental monitoring and management.

**Electronic supplementary material:**

The online version of this article (doi:10.1186/s12940-016-0104-5) contains supplementary material, which is available to authorized users.

## Background

Urbanization is an important social process underpinning the dynamics of human society, and it is especially impactful in the 21st Century. Generally, urbanization is accompanied by an increase in the proportion of urban to rural population, population growth in built-up areas; with urbanism referring to the urban lifestyle and its associated social and behaviour features [1]. Contemporarily, world urbanization has entered a special period with some new features including information cities or smart cities, multi-centred metropolitan areas, and further globalization involving the transmission of novel ideas and risk behaviours beginning in cities [2].

In China, urbanization has entered a period of accelerated development since the 1990s. Urban population growth in China is characterized by rural-to-urban migration. Nearly 40 % of people living in urban areas are migrants, with migrant populations numbering roughly 260 million [3]. To accommodate this significant immigration and population growth within cities, China’s urban area has increased rapidly, with large areas of farmlands converted to urban use. However,the urbanization process has progressed faster than economic growth since 2004, and it is now time to consider urbanization quality rather than a continuation of the spatial expansion from large scale “destroy and build” [4]. In March 2014, China unveiled the New-style Urbanization Plan (2014–2020) in an effort to steer the country’s urbanization onto a more human-centered and environmentally friendly path [5].

Despite numerous benefits originating from urbanization, as with other countries that are urbanizing rapidly, China also faces intensified resource scarcity and environmental degradation [6, 7]. Rapid urbanization impacts on natural and built infrastructure, environmental health and human wellbeing [8]. In this paper, we provide a systems overview of the relationship between urbanization, urban environmental change and health, and put forward possible solutions at national, local and individual levels based on evidence-based understandings of the links between urbanization and health in contemporary China.

## A systems understanding of the multiple ways in which urbanization impacts health

**Fig. 1:**
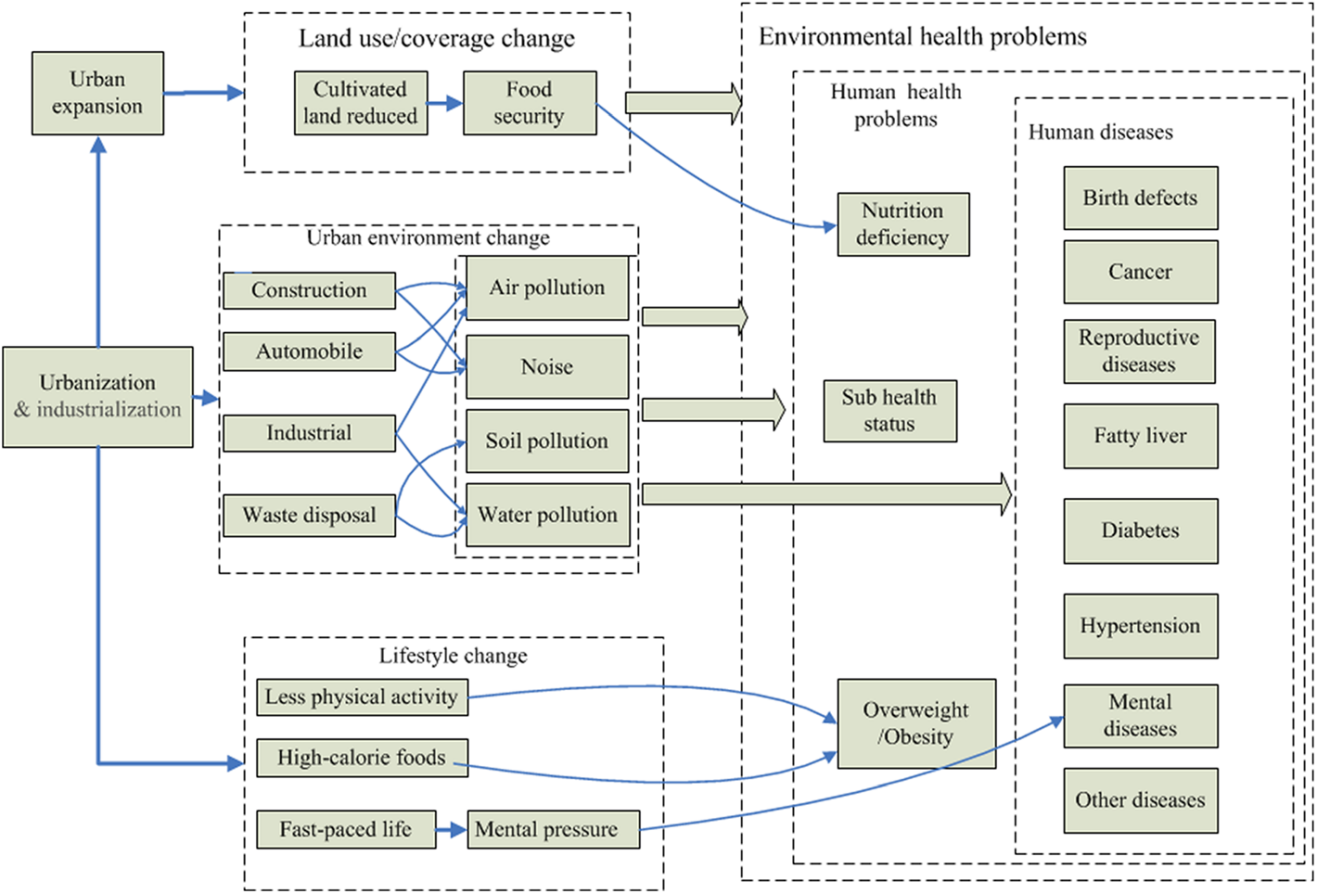
Relationship between urbanization, urban environmental change and public health [7, 86]

Urbanization and urban expansion result in urban environmental changes, as well as residents’ lifestyle change, which can lead independently and synergistically to human health problems. In particular, uncontrolled urbanization has been associated in some contexts with pollution, social isolation, overcrowding, changes in dietary and physical activity patterns, and inadequate service capacity for providing drinking water, sanitation and waste disposal, all of which raise the risk of harms to population health [9–11]. Adopting an ecological public health approach [12], which involves ‘the analysis of the composite interactions between the material, biological, social and cultural dimensions of existence’, Fig. 1 summarizes what is known about the mechanisms and pathways linking urbanization to health outcomes.

Figure 1 illustrates in particular how systemic changes in the environment as a result of urbanization pose numerous threats to human health. Rapid and often unplanned, urban growth is a source of environmental hazards, which have direct and indirect effects on human health. Urban expansion is one of the major driving factors of land use/coverage change in China, with extensive effects on local ecological systems through reducing biodiversity, air deterioration and contributing to water shortages [13]. Accelerated urbanization along with explosive economic growth has further worsened the shortage of agricultural land over the last two decades [14] with possible consequences for food security and nutritional deficiencies threatening the overall health status of the population. Reduced cultivated land places pressures to intensify agricultural production which depends on both the progress of agricultural technology and deeper dependence on usage of fertilizer and pesticides. Such inputs have repercussions for the availability of safe food, and also for the price of food, as fertilizer costs increase in line with oil prices.

Urban environmental change includes air pollution and noise caused by construction and transportation, and soil pollution and water pollution caused by waste disposal. Soil and water pollution can compound the problems and can cause human diseases directly [15]. Studies indicate that urban noise has adverse effects on human health, which may result in behavioural, psychological and physiological processes that pose risks to health. Noise exposure can impair the hearing system, producing temporary or permanent deafness [16]. China does not regulate the noise produced by construction activities and motor vehicles, to which urban residents are particularly exposed.

Urbanization not only transforms the urban environment, but encourages changes to people’s lifestyle, which is recognized by researchers as a key determinant of human health [17–19]. Growing numbers of people become reliant on automobile use and they can afford to buy computers; and high levels of car and computer use displaces more vigorous physical activity with passive activity. Fast food with high calories becomes more readily available as the numbers of time-poor but income-wealthier consumers grow, and is an increasing source of lunch food for urban workers. Reduced physical activity and high-calorie foods are the major contributing factors to the rise in body overweight and obesity world-wide, and China is no exception. Moreover, the fast-paced life in cities brings mental stress to residents, which like noise exposure can result in changed physiologic, psychological and behavioural processes [7].

## Major issues related to urbanization and health in contemporary China

### Health transition in China

China has undergone an epidemiological transition, shifting from infectious to chronic diseases in a much shorter time frame than many other countries [7]. Typically, chronic diseases have been considered a public health problem only in developed countries and among the elderly [20]. China’s epidemiological transition can be captured through changes in the causes of death in the adult population [7]. According to the Global Burden of Diseases research, the disease burden in China is dominated by cardiovascular diseases, lung cancer, chronic obstructive pulmonary disease, traffic injuries, and chronic disabilities such as musculoskeletal and mental disorders [21]. The burden of disease caused by individual behaviours and practices, such as diets low in fruit, high in sodium and low in whole grains, consumption of alcohol, smoking and insufficient physical activity, is steadily rising. Rates of smoking and physical inactivity are higher among urban residents than rural residents [22].

**Fig. 2:**
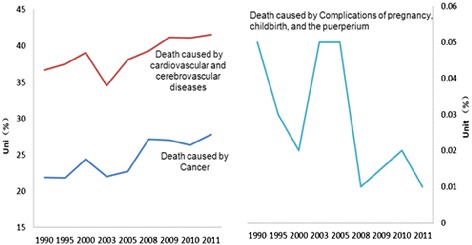
the proportion of death cause by cardiovascular and cerebrovascular diseases, cancer and complications of pregnancy, childbirth, and the puerperium. *Data source: 1. Death causes monitor dataset 2010; 2. Chinese Ministry of Health. Health statistic yearbook 2013

Specifically in urban areas, the proportion of mortality caused by cardiovascular and cerebrovascular diseases increased from 36.6 % in 1990 to 41.5 % in 2011, and that caused by cancer increased from 21.9 % in 1990 to 27.8 % in 2011. On the other hand the proportion caused by complications of pregnancy, childbirth, and the puerperium decreased from 0.05 to 0.01 %, shown in Fig. 2.

### Health risks from urban environment pollution

In addition to having cities with the worst air quality in the world, China’s cities are also facing serious water pollution problems. Environmental risk factors, particularly air and water pollution, are a major contributing source of morbidity and mortality in China [23]. Moreover, emissions of greenhouse gases from energy use are rapidly increasing. Global climate change will intensify China’s environmental health issues, with potentially catastrophic outcomes from major shifts in temperature and precipitation, as witnessed by the increasing extreme weather in recent years [23].

#### Urban air pollution

Urban air pollution is a worldwide problem responsible for a variety of risks to human health as reflected in a significant disease burden. According to WHO, almost 800,000 deaths and 6.4 million YLL (Years of Life Lost) was attributable to urban air pollution worldwide [24]. One of the most reported urban air pollutants is anthropogenic ambient particulate matter (PM), particularly ambient particulate matter with an aerodynamic diameter smaller than 2.5 μm (PM_2.5_). Atmospheric visibility has been found to anti-correlate well with PM_2.5_ concentration. Short-term exposure to PM_2.5_ will increase the risk of morbidity and mortality from cardiovascular and respiratory diseases [22].

Cities in China are among the most air polluted cities in the world, which has resulted from the rapid urbanization accompanied by increasing industrial development, energy consumption and urban traffic. Ammonium, nitrate, and sulphate carried by PM_2.5_ are the major contributor to regional haze in China, which are commonly found in coal burning and vehicle emissions [25]. In China’s urban areas, the number of motor vehicles has rapidly increased during the past several years. Emissions from these vehicles lead to the formation of photochemical smog within both city and adjacent regions. The economic cost of mortality and morbidity resulting from outdoor air pollution is a significant burden for Chinese cities. Of relevance to food security, the outflow of pollution from urban to rural regions has the potential to increase the concentrations of ozone in agricultural areas and depress crop yields.

Besides being a large emitter of greenhouse gases, China is also responsible for about 30 % of the global black carbon emissions from diesel engines and coal combustion. Black carbon is a key component of soot, and also a major contributor to climate change. Soot particles are a well-recognized health hazard associated with cancer and respiratory diseases [26].

Relevant to China, is the indoor combustion of solid fuels that releases large quantities of pollutants, which in turn increase the indoor concentrations of respirable particles and carbon monoxide to an extent more than ten times higher than globally set health standards. In larger cities, household coal use is declining but at the same time new sources of indoor air pollutants are increasing, such as formaldehyde and other chemicals released from building materials.

#### The health risks of water pollution

China is one of the few countries with inadequate water resources, for its population, having a fifth of the US water supply per head and less than a quarter of average world supply. Disturbingly from an equity perspective, China’s water resources are unevenly distributed. Water shortages compel some population groups to use unclean or polluted water sources, which can result in serious health effects, such as esophageal cancer [27]. Additionally, pollution has exacerbated water shortages in China. A large number of lakes and major rivers are severely polluted; only half of the major rivers and less than a quarter of the major lakes and reservoirs in China are suitable for drinking water after treatment [28]. Recent epidemiological studies indicate that some pollutants in drinking water, such as nitrite, nitrate, and chromium, are associated with cancers of the digestive system [29]. Toxic microcystins from the algal blooms in lakes and reservoirs can contaminate drinking water, and lead to rashes, diarrhea, nerve and liver damage, and even liver cancers. For example, the algal blooms in Taihu Lake have threatened millions of people who depend on this lake in Wuxi, Jiangsu province [30]. In addition to the chronic health effects of drinking polluted water, water pollution by industrial disaster could result in dramatic events, such as the explosion of the chemical plant on the upper reach of the Songhua River [31–33].

#### Climate change

Climate changes are recognized as exacerbating the existing environmental risk and related health effects in cities [34]. Increased frequency of extreme precipitation results in flooding. Widespread floods in 2007 led to more than 1200 deaths, and the loss of one million houses and large areas of crops. In recent years, urban flooding or waterlogging also occurred with increasing frequency, challenging the urban drainage system in China. During 2008 to 2010, about 62 % of the 351 cities experienced urban floods or water logging [35]. In addition to the direct fatalities during extreme rainfall, research indicates that populations that experienced flooding suffer from increasing mental disorders [36]. Moreover, heavy rains produce insect breeding sites, drive rodents out from burrows, and contaminate clean water systems, consequently increasing outbreaks of infectious disease [37].

Thermal stress is another issue which has attracted wide concern. Populations typically display an optimum temperature at which the death rate is lowest. Mortality rates rise at temperatures outside this comfort zone [38, 39]. Urban centers are often particularly affected because of the urban heat island effects [40]. Furthermore, increased temperatures can alter the biological habitat niche, promoting the survival, replication and transmission of waterborne pathogens, algae, and disease-carrying vectors; and, potentially increasing outbreaks of infectious disease, such as malaria, dengue, and West Nile virus [36, 41].

### The health consequences of an aging population

An aging population is present as a worldwide demographic shift, which presents both opportunities and challenges. With aspired long and healthy life, older people can be valuable social, cultural, economic and familial resources. On the other hand, aging populations may also mean higher demand for health care and social pensions, and a shrinking workforce [42].

China has more elderly people than any other country in the world, and the aging population has been increasing in size in recent decades. According to the 2010 census [43], the population aged over 65 is approximately 119 million, accounting for about 8.9 % of the total Chinese population. This proportion is estimated to reach 23 % in 2050 [3], when China’s proportion of aging population will match and even exceed that of many developed countries [44]. The percentage of the “very old”, people aged 80 years and above, has increased at a rate of 5.4 % annually since 1987 [45].

An aging population has profound effects on public health because of the growing number of elderly patients and the corresponding implications. Multiple morbidities are common in older people, with many being very costly, such as cardiovascular diseases, cancer, diabetes and chronic renal disease [46, 47]. Moreover, with increasing age so-called “geriatric impairments” in hearing, vision, cognition and mobility become increasingly prevalent [48].

Although reducing birth rates can reduce poverty and hunger, China’s one-child policy, introduced in 1979, and implemented for over thirty years, has resulted in an increase in older people and a decrease in younger workers. It is both urgent and necessary for China to solve its problems related to aging, both for public health and for sustainable economic and social development. In November 2013, a two-child policy was officially announced at the third plenary session of the 18th Communist Party of China Central Committee, which will allow families to have a second child if either parent is an only child [49]. However, as in much of the world, not all of the young people in contemporary China want to have many children. The latest surveys report that only 60 % of those who qualify to have two children under existing exemptions have actually expressed an interest in doing so [50].

### Migration and its health impacts

Characterized by large-scale rural–urban migration and expansion of built-up areas, China’s urbanization has produced many urban villages where large numbers of migrants live under poor living conditions with low access to health care. Migrant workers consistently underuse health services both in their original communities and in their destination cities, creating potential short-term and long-term health problems [51].

**Fig. 3:**
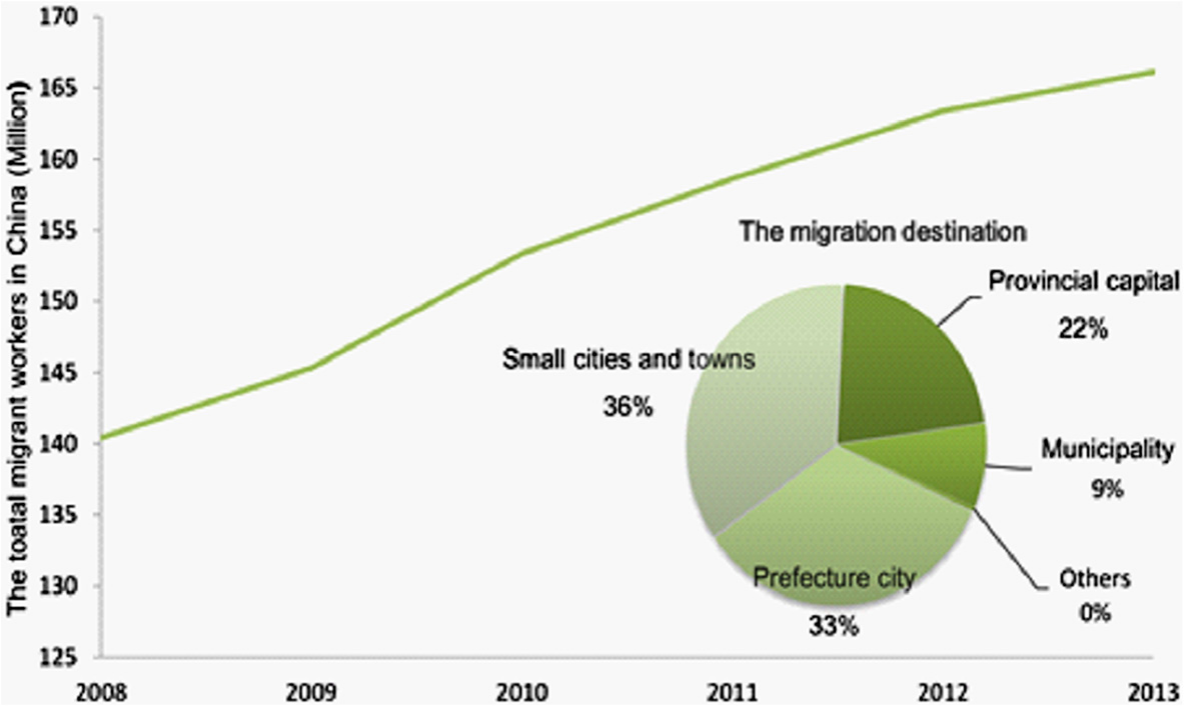
the total migrant works 2008–2013, and the migration destination in 2013 [50]

Rural–urban migration in China has accelerated with the rapid urbanization caused by socio-economic transformations at the beginning of this century [52]. According to the most recent national statistics released in 2014, the population of the rural–urban migrants in China increased from 140 in 2008 to 166 in 2013. Among these migrants, about 36 % migrated to small cities and towns, about 33 % migrated to prefecture cities, 22 % migrated to a provincial capital and about 9 % migrated to a municipality, as illustrated in Fig. 3.

In 2013, the average age of these migrants was 37.6, and only 6.7 % of them had college degree or higher. Over 67 % of these migrant workers had no experience of professional training for non-agricultural work. With an average monthly income of 2609 yuan (US$ 410), these workers had limited guarantees of rights and interests. Nearly 85 % of the migrant workers worked more than 44 h per week, 82.4 % had no medical insurance and 72.5 % had no injury insurance [53]. At Shenzhen in Guangdong province, researchers revealed that 62 % of migrant workers who reported illness did not visit a doctor [51]. In addition to the large number of the China’s adult migrants, children who are brought to the cities by their migrant parents or born in cities also have distinct health needs.

China launched a health-care reform plan in 2009 aiming to achieve universal healthcare coverage by 2020, “which will make up the health service gap between migrant workers and the original urban population [54]. According to a report based on the national monitor and survey of migrant workers in 2013, the proportion of migrant worker without injury insurance and medical insurance had decreased from 2008 to 2013, as show in Table 1. This means that an alarmingly high 117.6 million migrant workers were without injury insurance and an even higher 136.9 million were without medical insurance in 2013 [53]. Because China’s economy is so dependent on the large number of migrant workers [55], it would be prudent for the government to improve their living standards and health situation.

**Table 1:** Proportion of the migrant workers without injury or medical insurance from 2008 to 2013 [50]

	No injury insurance (%)	No medical insurance (%)
2008	75.9	86.9
2009	78.2	87.8
2010	75.9	85.7
2011	76.4	83.3
2012	76.0	83.1
2013	71.5	82.4

### The health consequences of low levels of regulation of the food supply

Food supply quality, including food safety, is of great importance for the public’s health in populous countries including China. With rapid urbanization, China’s food safety faces numerous challenges which are magnified by industrialization and modernization. The conversion of agricultural land to other uses, soil fertility decline and shortage of freshwater all limit the location and type of agricultural production that can be undertaken. Not only do soil and water pollution indirectly affect food production through making some areas not suitable for agriculture, but they contribute directly to increased health risks through contaminating agricultural products. At present, food safety policies are not integrated with soil and water pollution management policies [56] thus doing little to mitigate the risks. In the most recent decade, illegal additives have posed a growing food safety problem for China, leading to new public health hazards, loss of public confidence and social distrust of the food industry [57]. Gutter oil recycled from waste oil collected from restaurant fryer grease traps, drains, and slaughterhouse waste has emerged as a serious issue, exposing the shortcomings of China’s food safety assurance system [58].

China has struggled with food safety issues for years, and the government has shown determination to reform laws, establish monitoring systems, and strengthen food safety regulation. In 2003, the State Food and Drug Administration of China (SFDA) was founded to consolidate food and drug regulation. In June 2009, the Food Safety Law of the People’s Republic of China came into effect, which is the core regulation used to ensure the safety and quality of food and protect the health of consumers. Under the Food Safety Law, a food control management system has been established, including the State Council’s Food Safety Committee, the Ministry of Health (MOH), the Ministry of Agriculture (MOA), the Administration of Quality Supervision, Inspection and Quarantine Department (AQSIQ), the Industry and Commerce Department (IAC) and the State Food and Drug Administration Department (SFDA). These central government agencies take the responsibility of administrative management at the national level, while at the local level, departments of health, agriculture, quality control, industrial and commercial, and food and drug are required to coordinate with each other to implement Food Safety Law [59]. Several data management systems related to food safety are running online now, such as the Animal Labelling and Disease Traceability System and the National Monitoring and Control Plan on Animal Drug Residues in Animals and Animal Products [57]. However, there remain problems and gaps in the supervision and administration of the overall food safety system. The relationship between central governments and local governments requires further clarification, and in particular the responsibility of ministries and governments at the local level needs to be more clearly defined. Media, consumers and third parties could also play a more important role in holding the management authorities to account for their activities. While significant advances have been made, food safety regulations and their coordination can be further strengthened [60].

## Posing multi-level solutions: national, local and individual levels

**Fig. 4:**
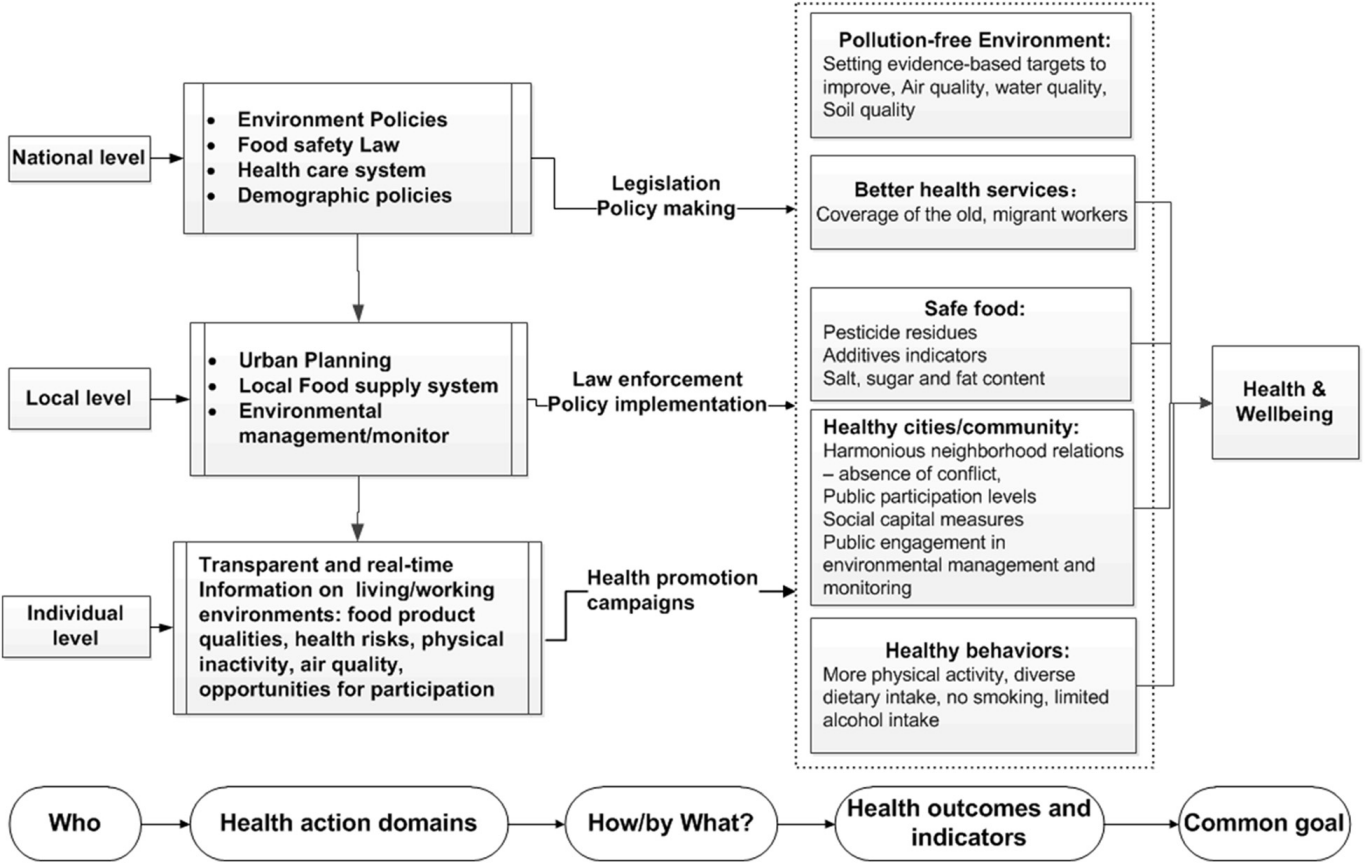
Strategy at national, local and individual level for health and better life

A multi-level understanding of the relationship between the features of China’s urbanization, urban environmental change and risks to public health provides the basis for identifying interventions at national, local and individual levels. Following from the evidence provided in this review, attention needs to be focused on the following key public health domains: a pollution-free environment, a safe and diverse food supply, a health system that addresses the needs of hard-to-reach groups, planning for healthy cities/communities, and health behaviour change education. Concerted and coordinated action by the central government, the local government and the public in each of these areas could advance the goal of health and wellbeing for all of China’s citizens. Figure 4 describes the major categories of health promoting activity alongside the administrative or governance level which has major responsibility for the activity, the types of regulatory or change mechanism which would be involved, and examples of the types of measurable indicators of achievement towards the shared goal of improved health and well-being. In the sections that follow we provide more concrete examples at the three levels.

### Strategy at national level

Through its control of national legislation and policy instruments, the central government is best placed to enact strong environment policies, food safety laws and regulations, review the coverage of the health care system, and make gradual adjustments to demographic policies. Evidence suggests that it is urgent to replace the current quantity-oriented environment campaign targets with targets emphasizing ecosystem function, as discussed in the Jianguo Wu’s paper [61]. Urban ecologists recognise that healthy cities and a healthy planet are part of the same system, and that it will not only be necessary to reduce the resource throughput of cities but new socio-technical systems are required that are capable of recycling wastes and capturing pollution [62]. In China, there are calls to reduce the subsidies allocated to high carbon content fuels [62], and to incorporate environmental and health co-benefits into climate policies [63]. Regarding PM_2.5_, the pursuit of low-carbon electricity generation would be cost-effective if the costs of pollution-related health problems under existing energy generation technologies are taken into account [64]. Forging links between energy and water policy and overall development planning are also under discussion, as it is in United Nations agencies with the UN declaring 2005–2015 as the Water for Life decade [65], and it is expected that this initiative will drive more effective multi-sector action. The next step would be to create links to urban agricultural and food policy. In one meta-review of studies which examine sources of pollution and chemical contamination of city-grown foods across China, polycyclic aromatic hydrocarbons recur as a major contaminant, which is in part due to coal fired combustion [66]. Based on the Food Safety Law, China has established a food safety insurance system, but more efforts at responsibility clarification in relation to policy insurance are needed. In this regard, China’s food safety laws could be influenced by the One Health movement which is based on the principle that healthy agricultural ecosystems produce healthy animals and together they produce healthy populations [67]. To establish a universal coverage basic healthcare system, providing affordable basic health care to everyone in the country, the Chinese central government launched a national health reform plan in 2009. Two years later, China achieved basic healthcare coverage for over 90 % of the population, which was realized through a universal health insurance system [68]. There is still room for improvement in terms of health equity, especially in relation to health care accessibility and affordability for the aging population and the migrant workers. Finally, population policies need to be redesigned for the inevitable trend towards an aging population and the profound effects of the one-child policy for labour market functioning and national prosperity. The challenge for the Chinese government is to fine-tune support for mobile populations in ways that are environmentally sustainable, culturally acceptable and deliver the best outcomes for health and well-being of the mobile population.

### Strategy at local level

As the policy implementers, local governments can shape city eco-systems and population health by improving urban planning [69], establishing local food supplies which are safe and offer sources of diverse nutrition [70], and enforcing environmental monitoring and management [71]. Each local planning agency could forge close partnerships with urban health and wellbeing planners, thereby moving the public health function into local authorities. Small food producers can be empowered to learn from one another about how to improve food safety through improving agricultural environments [72]. Local government could become members of the Global Healthy Cities movement to share ideas about how urban planning can be used to shape health promoting physical and social environments [73], and take advantage of research on urban environmental health and sustainability developed by the Healthy-Polis consortium. In this way, cities can foster simultaneously sustainable development and public health. Health-centred local planning would involve focusing urban design on more open green space and outdoor recreational facilities, building design for more physical activity and high temperature mitigation [74]. Through the provision of more diverse and easily accessible cultural and exercise opportunities, the building of harmonious communities or neighbourhoods can result and thus reduce urban-related mental stress and aloneness. In terms of advancing healthy dietary behaviours, local authorities can support local food systems through allocating space to urban gardens and agriculture. There are some notable examples where local authorities are combining agro-tourism with sustainable urban food systems which involve waste recycling [75]. Local government can also create physical spaces for people to shop for healthy foods including safeguarding or providing spaces for fresh food markets. Access to fresh food markets has been shown in other Asian countries to be important for low income communities given that plant based foods are generally cheaper in traditional retail formats than in supermarkets [76]. In addition, local government can use planning tools to provide food environments which encourage people to eat together, rather than alone. In many cultural contexts, eating together can have health benefits [77]. In relation to food safety, local government is the relevant authority for enforcing food safety laws. In particular, local government should consider leading the reconstruction of the local food supply system for the following reasons: Firstly, much food contamination harmful to health takes place during the storage and transport stages [57], which could be reduced through establishing a local food supply system. Secondly, a local food supply system could provide more fresh fruits and vegetables which are good for health. Thirdly, participation in the labour-force at urban farms could increase physical activity and reduce mental stress. Labour force participation also brings household incomes which in turn can be allocated to healthier diets. China is well-placed to consolidate its encouragement of household food production and could draw upon experience with urban agriculture elsewhere [77]. With the development of the information technology and the internet, local government could introduce action research methods involving local residents combined with food or green space mapping using GIS technologies to monitor and manage the urban environment. The International Centre for Sustainable Cities has an urban greening partnership program which has supported local authorities in several Asian cities to work with residents on environmental improvements including using urban spaces for food production [78]. China has long been regarded as a centralized society where the public has little influence on decision-making; and there is a view that a lack of public participation in environmental monitoring and environmental management make responses less efficient. Given that urban residents directly suffer urban environmental pollution they have a stake in becoming more active in alerting authorities to immediate harmful environmental pollution. Fortunately, there is a precedent: the recent popular struggle against PM_2.5_ has opened a door to public participation for addressing environmental issues in China [79].

### Strategy at the individual level

Individuals too should be encouraged to take responsibility for their health within the context of a supportive regulatory and policy environment, as outlined above. According to the Global Burden of Diseases data base, much of the disease burden in China’s urban areas is associated with individual behaviours and practices, such as diets low in fruit, high in sodium and low in whole grains, consumption of alcohol, smoking and insufficient physical activity, which is steadily rising in China [21]. Before they can make behavioural choices however, urban citizens require facts about their environment and food supply. Just as consumers in other nations are demanding more information on food producers, production methods and product quality of their food, so Chinese consumers need access to such information [80]. There is also a case for improving nutrition literacy through government campaigns, backed up by fiscal and regulatory measures in relation to food advertising [81]. Health promotion activities aimed at individuals will be most successful if they take into account household resources, education and local area facilities and services [82]. Possibly the most important health promoting mechanism is investments in high levels of education, labour market opportunities and housing which has clean water, electricity and has good access to public transport. Under this set of conditions, individuals have greater choices available to them to adopt healthy lifestyles. This is the logic which has underpinned the Healthy Cities program since it began in 1986 [83], with the China Hong Kong chapter sponsoring the most recent Conference of the Global Alliance for Healthy Cities.

## Conclusions

Urbanization in China, accompanied by increasing economic growth and consumerism, overwhelms and marginalises numerous ecological and social issues, which in effect lead to problems in public safety, public health, and social equity. Other than learning from those developed countries which have experienced rapid urbanization, China is also challenged by an unprecedented complicated situation, because of a large and aging population, significant environmental degradation brought about by rapid industrialization, and frequently reported food safety issues. Based on systems thinking, and scientific evidence of the links between broad trends, including public health risks, it is possible to propose solutions at national, local, and individual levels. Most importantly, the central and local governments and the public need to work together to advance the common goal of urban health and human wellbeing. At the national level, the central government should act on current environment policies, food safety laws, and the health care system, while incrementally adjusting demographic policy. At the local level, the local government is well-placed to shape the city ecology towards healthy life by using urban planning, establishing a local food supply system, and enforcing environmental monitoring and management. Within the context of government regulations and investments in improvements to urban infrastructure, individuals should be encouraged through health education to take responsibility for their health behaviours. Moreover, they should be encouraged to participate in environmental monitoring and management by using portable monitoring equipment or just smartphone app. Classified as an upper-middle-income country by the World Bank in 2011, China could play a more important role in global health [84, 85]. This leadership potential could be strengthened through its institutions promoting the type of systems thinking outlined in this paper, which links an understanding of urbanization, demographic and socio-economic trends and health at the national, local and individual levels.

## Additional file

Additional file 1:
**Peer review reports.** (PDF 411 kb)
